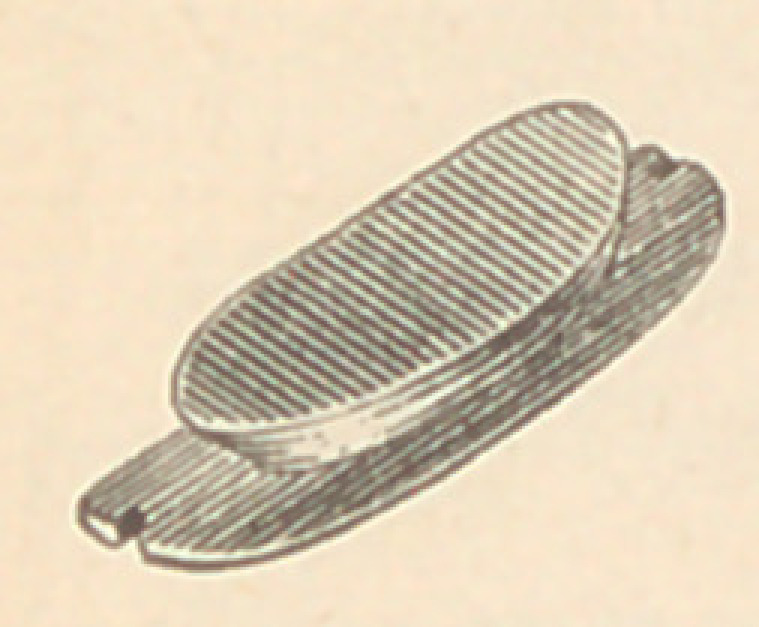# Current News and Opinion

**Published:** 1886-06

**Authors:** 


### Current News and Opinion.

### NUTRITION FOR INFANTS AND INVALIDS.

In the study of infantile nutrition the chief question is the fitness in quality and composition of the food for the purpose intended.

Human milk is the typical food for the race. We find on examination that it is composed of several alimentary principles :

First.—A nitrogenous substance (casein) and small quantities of other forms of albuminous matter.

Second.—Fatty matter (butter).

Third.—A carbo-hydrate (lactose or milk sugar).

Fourth.—Salts and inorganic matter.

It is especially necessary in a food designed for infants and invalids that the nearest possible approach to the above standard be realized. Cow's milk varies so much from this standard that it is necessary to combine with it other substances that will supply its deficiencies. The most notable differences are in the greater amount of milk sugar and the smaller amount of casein which human milk contains.

For many years the best medical authorities have recommended the use of milk sugar in food for infants, and with the happiest results. It is recommended because, as Prof. Kuss says in his Physiology (p. 301), "the principal element in woman's milk is the sugar of milk." Not only does it give a pleasant taste, but it has been found by Dr. Ruschenberger to have an excellent effect, even in extreme irritability of the stomach. Dr. C. H. Routh, in his work, "Infant Feeding and its Influence on Life," says : "Sugar of milk allays morbid irritation, and will often check diarrhœa." It will thus be seen to be not only highly nutritious, but at the same time has medical properties that will prove of great value for the large class of infants that are predisposed to irritability of the stomach and bowels.

Cane sugar, which is so universally used to sweeten food for infants, and often in excessive amounts, on the contrary has a great tendency to produce at once fermentation in the stomach, and break up into carbonic acid and alcohol, and thus augment instead of allay the irritation. It often produces gastric catarrh and diarrhœa, and oftentimes results in constipation, with irritation of the mucous membrane, as is often indicated by sore mouth, etc.

The fermentation resulting from milk sugar (which takes place very slowly) is the lactic fermentation, producing lactic acid—the true acid of digestion.

Food, to be capable of supporting life, must contain three kinds of substances in due proportion :—

1. Plastic or nitrogenous matter, to nourish the fleshy or muscular parts of the body.

2. Calorifiant or combustible matter, i. e., carbo-hydrates, to supply the respiratory organs, to preserve animal heat, and provide fat for the body.

3. Mineral matters or salts, for the growth of the bones and to hold in chemical union, combination, and action the solids and liquids of the body.—*Extract.*

### UNIVERSITIES OF EUROPE.

The following is a list of the Universities of Europe, as compiled by a prominent dentist in Germany, and transmitted for publication in the INDEPENDENT PRACTITIONER, by Dr. Louis Ottofy, of Chicago.

AUSTRO-HUNGARY: Agram, Budapest, Czernowitz, Graz, Innsbruck, Klausenburg, Cracow, Lemberg, Prague, Vienna.

BELGIUM: Brussels, Gand, Louvain, Liége.

DENMARK: Copenhagen.

FRANCE: Angers, Lille, Lyons, Paris, Poitiers, Toulouse.

GERMANY: Berlin, Bonn, Breslau, Erlangen, Freiburg, Giessen, Gœttingen, Greifswald, Halle, Heidelberg, Jena, Kiel, Kœnigsberg, Leipzig, Marburg, Muenchen, Rostock, Strassburg, Tuebingen, Wuerzburg.

GREAT BRITAIN: Aberdeen, Cambridge, Dublin, Durham, Edinburg, Glasgow, London, Oxford, St. Andrews.

ITALY: Bologna, Cagliari, Catania, Genoa, Macerata, Messina, Modena, Naples, Padua, Palermo, Parma, Pavia, Pisa, Rome, Sassari, Siena, Turin.

NETHERLANDS: Grœningen, Leiden, Utrecht.

NORWAY: Christiania.

PORTUGAL: Coimbra.

RUSSIA: Charkow, Dorpat, Helsingfors, Kiev, Odessa, Petersburg, Warsaw, Vilna.

SPAIN: Barcelona, Granada, Madrid, Oviedo, Salamanca, Santiago, Saragossa, Seville, Valencia, Valladolid

SWEDEN: Lund, Upsala.

SWITZERLAND: Basle, Berne, Geneva, Zurich.

---

### THE AMERICAN DENTAL ASSOCIATION.

The votes of nearly all the members have been received. A majority of the votes cast are in favor of Chicago over all other places, and a very large majority pledge their attendance if the meeting shall be held in Chicago. But in deference to the minority, and for the sake of harmonizing all differences, as chairman of the Executive Committee of arrangements, I hereby, with the consent of my colleagues, announce the next place of meeting to be at Niagara Falls, August 3

State and Local Dental Societies should remember that every local society which has adopted substantially the code of ethics of the American Medical Association is entitled to one delegate for every five members Appoint your delegates soon, and let all unite in making this the largest and most profitable meeting of the Association yet held.

Information concerning hotel and railroad rates will be given later.

J. N. CROUSE, 2101 Michigan Ave.,
Chairman of Ex. Com.

### NATIONAL DENTAL ASSOCIATION.

The next regular biennial meeting of the National Dental Association of the United States of America will be held at Washington, D. C., July 27, 28 and 29, 1886.

OFFICERS.

*President*—R. B. Winder, Baltimore, Md.
*First Vice-President*—John B. Rich, New York.
*Second Vice-President*—V. E. Turner, Raleigh, N. C.
*Third Vice-President*—W. W. Ford, Macon, Ga.
*Fourth Vice-President*—E. Parmly Brown, Flushing, N. Y.
*Fifth Vice-President*—J. H. Coyle, Thomasville, Ga.
*Secretary*—R. Finley Hunt, Washington, D. C.
*Treasurer*—H. B. Noble, Washington, D. C.

Very respectfully, R. Finley Hunt,
Sec. N. D. A. U. S. A.

---

### THE AMERICAN MEDICAL ASSOCIATION.

The thirty-seventh annual session of the American Medical Association held in St. Louis, was a large and harmonious meeting. As was anticipated, no change in the organization of the International Congress was made, and the matter stands as it did before the meeting. Dr. N. H. Davis, of Chicago, was elected President of the Congress, in place of Dr Austin Flint, deceased. Dr. E. H. Gregory, of St. Louis, was elected President of the Association, the next meeting of which will be held in Chicago. Of the Section of Oral and Dental Surgery, Dr. J. S. Marshall, of Chicago, was elected Chairman, and Dr. E. S. Talbot, of Chicago, Secretary.

---

### A SPECIMEN OF COMPARATIVE DENTAL PATHOLOGY.

Among the playthings of a little child was found what seems to be the tooth of a cat. Nothing is known of the history of this tooth, but it bears strong evidence of having been a troublesome member. Upon one root, as will be seen in the cut, the entire surface appears to be roughened by exostosis from the apex almost to the cervix. Or are these the marks of the mysterious pyorrhœa alveolaris? The other root is atrophied to a marked extent. Poor pussy, she had her dental troubles as well as we, whatever may have been the exact symptoms of her case.

Have we here a possible cause for some of those nocturnal complaints that we hear at times with so much regret?

Henry N. Dodge, M. D., D. D. S.,
Morristown, N. J.

---

### MARRIED.

On the 24th of April, at the Church of the Holy Communion (Episcopal), New York City, Dr. Lawrence Vanderpant, of Orange, New Jersey, to Margaret Ellis, formerly of London, England.

### A MODIFIED MATRIX.

It is sometimes extremely difficult to satisfactorily fill cavities upon the posterior surface of second molars, when the third molar is only partially erupted.

Dr. J. A. Swasey, of Chicago, meeting with such a case, took an ordinary Jack's matrix and placed a button of soft solder upon its posterior surface. This was shaped with a file, and a groove made around it, as is represented in the accompanying cut. A hole is cut in the rubber dam, and it is adjusted in the groove. Holes are cut for the second and first molars, and the matrix firmly placed between the second and third molars. The rubber dam is thus easily adjusted and held in place, there being no necessity for its enveloping the third molar. It is a very ingenious and effective device, and one easily made by any dentist.

---

### DR. HERBST COMING.

Dr. W. Herbst, the originator of the Herbst system of filling teeth by rotary motion, will arrive in New York on or about June 25th. He will give clinics before the First District Dental Society at White's Dental Depot, Broadway and Ninth St., in the afternoon, and at the New York College of Dentistry in the morning, during the first week in July. Members of the dental profession are invited to attend and satisfy themselves concerning the merits of the rotary system.

---

### ODONTOGRAPHIC SOCIETY.

The Odontographic Society of Philadelphia, at the twenty-third annual meeting, re-elected Drs. Jos. R. C. Ward, President; C. A. Kingsbury, 1st Vice-President; Chas. E. Pike, 2d Vice President; Jno. W. Wunderlich, Treasurer; Chas. E. Graves, Rec Secretary; Alonzo Boice, Cor. Secretary; S. J. Dickey, Curator; C. J. McCartney, Librarian; Thos. C. Stellwagen, L. Ashley Faught, and Wm. A. Green, Ex. Committee.

CHAS. E. GRAVES, D. D. S., Rec. Secretary.

---

### FIFTH DISTRICT DENTAL SOCIETY.

The eighteenth annual meeting of the Fifth District Dental Society of the State of New York, was held at Stanwix Hall, Rome, N. Y., Tuesday and Wednesday, April 13 and 14, 1886. Five new members were received. The officers elected for the ensuing year are as follows :

*President*—G. L. Curtis, Syracuse.
*Vice-President*—C. H. Bennett, Waterville.
*Recording Secretary*—C. J. Peters, Syracuse.
*Correspondent*—B. T. Mason, Phœnix.
*Treasurer*—A. R. Cooke, Syracuse.
*Librarian*—A. Retter, Utica.

### MINNESOTA STATE BOARD.

A regular meeting of the Minnesota State Board of Dental Examiners will be held Saturday, July 25th, 1886, at St. Paul (immediately after the Minnesota State Dental Society), for the purpose of examining applicants to practice in the State of Minnesota.

J. H. MARTINDALE, Sec'y.

---

SPONGE-GRAFTING—WHAT BECOMES OF THE SPONGE?—Dr. John C. Cotton, of Meadville, Pennsylvania, asks this question. By some it is claimed that the sponge, after forming a temporary trellis or support for the new granulations, when they are able to support themselves, becomes digested and absorbed. By others it is maintained that the sponge itself becomes organized and forms a constituent of the new growth. Still others contend that the sponge simply affords a proper covering for the new granulations, and by its presence stimulates their growth, and when this is accomplished, it is thrown off as effete matter. Dr. Cotton lays down the following deductions: 1. The sponge is not organized, but simply forms a support for the new granulations, which shoot up and interlock or coalesce through its meshes, until they become sufficiently strong not to require an artificial prop, when it is digested. 2. The Sponge sometimes does rise in the wound, but in such cases it is the result of accident, such as want of sustained equable compression, etc. When perfectly successful it should adhere to the base of the cavity, and be completely enveloped in the new structure. 3. Epithelial cells are not always derived directly from an epithelial margin, but may be transmitted through a medium, probably pus.—*Provincial Med. Jour.*

---

PROFESSOR HUXLEY, in a certain debate on smoking among the members of the British Association, told the story of his struggles in a way which utterly put the anti tobacconists to confusion. "For forty years of my life," said he, "tobacco had been a deadly poison to me. [Loud cheers from the anti-tobacconists.] In my youth, as a medical student, I tried to smoke. In vain! At every fresh attempt my insidious foe stretched me prostrate on the floor. [Repeated cheers.] I entered the navy. Again I tried to smoke, and again met with defeat. I hated tobacco. I could have almost lent my support to any institution that had for its object the putting of tobacco smokers to death. [Vociferous cheering.] A few years ago I was in Brittany with some friends; we went to an inn; they began to smoke and look very happy, and outside it was very wet and dismal. I thought I would try a cigar. [Murmurs.] I did so. [Great expectations.] I smoked that cigar—it was delicious! [Groans.] From that moment I was a changed man, and now I feel that smoking in moderation is a comfortable and laudable practice, and is productive of good. [Dismay and confusion of the anti-tobacconists. Roars of laughter from the smokers.] There is no more harm in a pipe than there is in a cup of tea. You may poison yourself by drinking too much green tea, and kill yourself by eating too many beefsteaks. For my own part, I consider that tobacco, in moderation, is a sweetener and equalizer of the temper." [Total rout of the anti-tobacconists, and complete triumph of the smokers.]—*Medical and Surgical Reporter.*

THE SUCCESSIVE ANNUAL REPORTS of the Iowa State Board of Dental Examiners, forwarded by the Secretary, Dr. W. P. Dickinson, show a steady growth in the organization of the profession in that State. After all, it is the moral support of the people, which is gained by complete organization, that is the greatest benefit derived from legislation. There are few men who alone are competent to conquer the respect of all the world, but a thorough organization of even the weakest will bear down all before it.

The reports also show the benefits of legislation in another way. When the Iowa State dental law was passed, there were but twenty-three dental graduates in the State. Now there are about one hundred.

---

THE SOUTHERN CALIFORNIA PRACTITIONER is a very bright and readable journal. It shows excellent judgment, too—far above that of the average medical man—when it gives a list of the members of the Southern California Odontological Society and then says:

"The members of the medical profession are often asked: 'Who is the best dentist,' and we recommend them to refer to the above list, because it contains the names of bright, energetic, professional dentists. Quacks, in all professions, shun scientific societies."

---

A COMMITTEE OF FIVE MEMBERS was appointed at the late meeting of the Illinois State Dental Society to take into consideration and to report, if possible, some feasible means to enable Dr G. V. Black to devote his whole attention to histological and pathological research. Such work cannot be perfectly done when one is obliged at the same time to conduct a private practice. The general idea seemed to be that, to carry on successful original scientific observations, the observer must be enabled to give to it his whole time. The object of the movement is a noble one

---

DR. J. S. WALTER, of Rochester, who has for many years been engaged in the practice of dentistry in Western New York, has gone—

> "Where rolls the Oregon, and hears no sound
> Save his own dashings."

His removal leaves but one of the brothers Walter in practice in this State. Dr. Walter's new address will be Ashland, Oregon.

---

A MAN whose opinions are not attacked is beneath contempt. Every real thought on every real subject knocks the wind out of somebody or other.

I find the great thing in this world is not so much where we stand, as in what direction we are moving.

Controversy equalizes fools and wise men in the same way—and the fools know it.—*Oliver Wendell Holmes.*

---

THE AMERICAN SOCIETY FOR THE ADVANCEMENT OF SCIENCE meets in Buffalo, in August of this year. This society has, ever since its organization, held its every tenth meeting in the Queen City of the Lakes, and this is the decennial year.

The advent of cocaine as an anæsthetic was heralded with the utmost degree of enthusiasm, and its marvelous effects as a pain obtunder were pictured in glowing colors. Now, however, it is to be observed that the enthusiasm has spent its force, and to the enthusiast the picture has lost much of its brilliancy. Imagination has played a prominent part in both directions, and *imagination* wields powerful influences for good or evil among mankind.

---

A lady, with her head bandaged, rushed into her dentist's office and exclaimed: "Doctor, I cannot stand it any longer. These artificial teeth you made for me cause the most terrible agony."

"Well, Madam," was the answer, "what would you have? I could not imitate nature any better."

---

A Box Containing several pieces of prehistoric Etruscan dentistry was exhibited at the Kansas and Illinois State Dental Societies. The specimens were secured by Dr. J. G. Van Marter, of Rome, Italy, from lately discovered Etrurian graves, and were forwarded by him to the Independent Practitioner. They are the earliest pieces of dental work extant.

---

At the Late Meeting of the Kansas State Dental Society, R. I. Pearson & Co., of Kansas City, made a remarkable show of Dental materials. Their line of the S. S. White Manufacturing Co.'s goods must have been well nigh complete. Certainly, it would have done credit to that company, had it been their own display.

---

James Vick, of Rochester, N. Y., may well claim to be the monarch of the floral world. It is doubtful if there exists any other man who is so extensive a dealer in seeds and shrubs. If any one wants a floral variety, let him send to Mr. Vick, with the certainty that he will receive only seeds that are fresh

---

The Weekly Medical Review, of St. Louis, published a daily bulletin containing the proceedings of the American Medical Association. It was an instance of decided enterprise. We beg to acknowledge the courtesy of the publishers in sending us copies.

---

Dr. Thomas W. Evans, of Paris, has been honored by the king of Sweden with the decoration of the "Order of the Polar Star," for a successful operation of oral surgery. So reports the *London World.*

---

Dr. W. Storer How is sending out blank forms for recording the exact symptoms and conditions attending all diseased conditions of the mouth. They are to be used for the benefit of the section of Etiology and Physiology, in the American Dental Association.

---

The Third Annual Meeting of the National Association of Dental Faculties will be held at Niagara Falls, Wednesday, August 4th, at 3 p. m.

H. A. Smith, Secretary. C. N. Peirce, President.

H. Boyle Runnels, in the *Bristol Medico-Chirurgical Journal*, says it is his experience that *every* case of so-called neuralgia, not due to a nerve implicated by a growth, carious bone, or a scar, is the result of dental irritation. Dr. J. M. Ackland, in the same journal, would add general debility as sometimes a cause, and ascribe all the rest to dental troubles.

---

The St. Louis Weekly Review, during the meeting of the American Medical Association, gave a reception and dinner to the editors of the medical profession. The evening so spent was said to have been a very enjoyable one, and the courtesy and hospitality of the *Review* was highly appreciated. The editor of this journal returns his appreciative thanks for an invitation to be present.

---

The Southern Dental Association will meet at Nashville July 27, 1886, and will continue four days. Preparations are being made for a large meeting, and from the intelligence so far received it is believed that it will be one of unusual interest. Members of the profession from every locality are cordially invited to be present.

---

Dr. G. V. Black dissents most emphatically from the time-honored teachings of dental anatomy concerning the function and structure of the peridental membrane. His views are set forth in the report of the Illinois State Dental Society, in this number.

---

A Death Certificate returned to the proper authorities by a Cincinnati physician gives the cause of death as follows: "She dide with Liver dease & New Monei."

---

Important from Hot Springs.—A friend of ours went to the Springs for change and rest. The waiters got his change and the hotels the rest.—*Weekly Medical Review.*

---

At the Meeting of the Illinois State Dental Society, Mr. C. Geo. Crowley, of New York, was elected an honorary member in recognition of his able literary work done for the profession.

---

The S. S. W. D. M. Co. put up red and blue litmus cut in small squares and contained in bottles. They are very convenient for testing for acids and alkalies.

---

Dr. W. D. Miller was elected an honorary member of the Illinois State Dental Association by acclamation, and with great acclaim.

---

The Minnesota Hospital Medical College has established a Spring course adapted to the instruction of dental students.

---

Dr. B. Merrill Hopkinson has again resigned his position as assistant demonstrator in the University of Maryland, Dental Department.

---

Dr. Carver, the marksman-dentist, prefers plugging glass balls with lead to plugging teeth with gold.

## Figures and Tables

**Figure f1:**
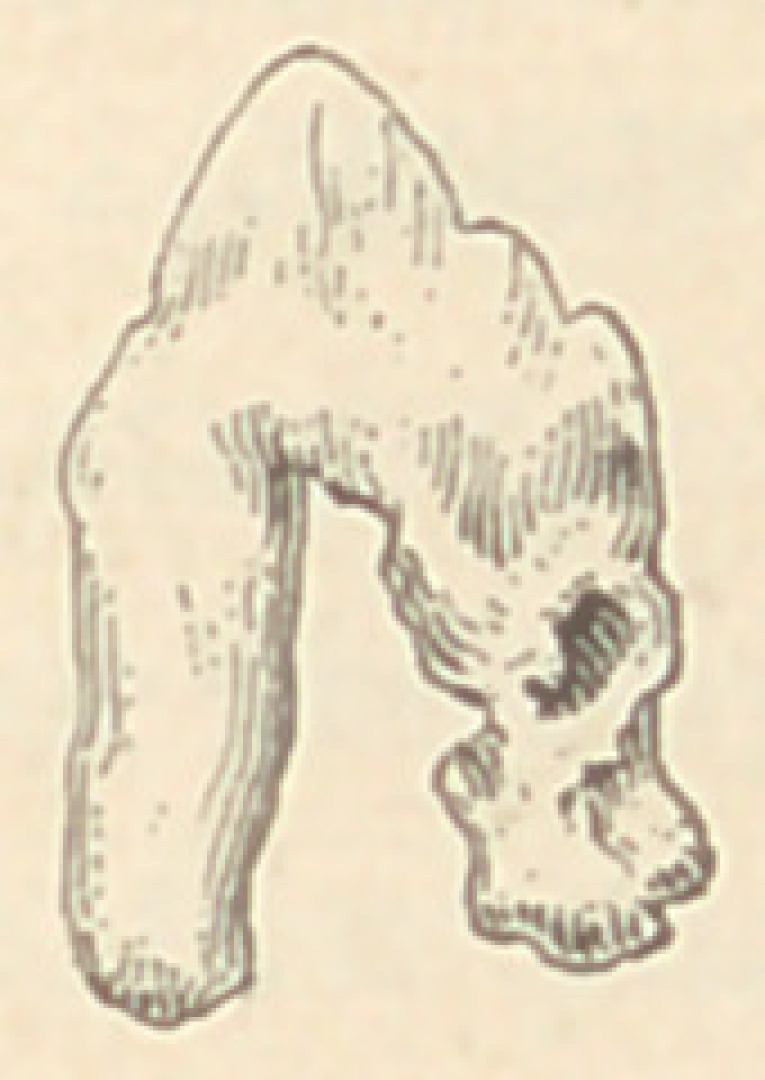


**Figure f2:**